# Antihypertensive medication needs and blood pressure control with weight loss in the Diabetes Remission Clinical Trial (DiRECT)

**DOI:** 10.1007/s00125-021-05471-x

**Published:** 2021-05-31

**Authors:** Wilma S. Leslie, Eman Ali, Leanne Harris, C. Martina Messow, Naomi T. Brosnahan, George Thom, E. Louise McCombie, Alison C. Barnes, Naveed Sattar, Roy Taylor, Michael E. J. Lean

**Affiliations:** 1 Human Nutrition, School of Medicine, Dentistry and Nursing, College of Medical, Veterinary & Life Sciences, University of Glasgow, Glasgow, UK; 2 Robertson Centre for Biostatistics, Institute of Health and Wellbeing, University of Glasgow, Glasgow, UK; 3 Human Nutrition Research Centre, Institute of Health & Society, Newcastle University, Newcastle upon Tyne, UK; 4 Institute of Cardiovascular and Medical Science, University of Glasgow, Glasgow, UK; 5 Magnetic Resonance Centre, Translational and Clinical Research Institute, Newcastle University, Newcastle upon Tyne, UK

**Keywords:** Blood pressure, Remission, Type 2 diabetes, Weight management

## Abstract

**Aims/hypothesis:**

Our aim was to evaluate the safety and efficacy of a planned therapeutic withdrawal of all antihypertensive and diuretic medications, on commencing a formula low-energy diet replacement, targeting remission of type 2 diabetes.

**Methods:**

Post hoc analysis of changes in BP, antihypertensive medication prescriptions and symptoms during the initial total diet replacement phase was performed in the intervention arm of the Diabetes Remission Clinical Trial *(n* = 143) and in the subset *(n* = 69) who discontinued antihypertensive medications at the start of total diet replacement. The Counterweight-Plus total diet replacement provided about 3470 kJ/day (830 kcal) with automatic reductions in all nutrients, including sodium, to achieve marked negative energy balance and rapid weight loss over 12–20 weeks, with regular BP monitoring and an antihypertensive reintroduction protocol based on current clinical guidelines.

**Results:**

Of 143 intervention group participants who commenced total diet replacement, 78 (55%) were on treatment for hypertension at baseline. The overall mean BP fell significantly from the start of total diet replacement (week 1) and was significantly lower at week 20, after total diet replacement finished, and also at 12 and 24 months. Of the 78 participants previously on treatment for hypertension, 65 (83%) stopped all antihypertensive and diuretic medications as per protocol, and four (5%) stopped some drugs. These 69 participants experienced no immediate (within the first week) change in BP, but their mean BP fell significantly from 9 weeks. No excessive rises in BP were recorded in individuals, but antihypertensive medications were reintroduced during total diet replacement to manage raised BP for 19/69 (27.5%) participants, mostly within the first 3–7 weeks, despite some weight loss. Reintroduction of antihypertensive medications was necessary for 5/19 participants previously on one drug, and for 14/19 previously on two or more drugs. Of the 69 who stopped antihypertensives, 19 (28%) remained off medications at 24 months. Among the 53 participants who achieved sustained remissions of diabetes at 24 months (with a mean weight loss of 11.4 kg), 31 had been previously treated for hypertension. Twenty-seven stopped medication at baseline, and 15/27 required reintroduction of antihypertensive medications. Mild to moderate dizziness, suggesting some postural hypotension, was reported during total diet replacement by 51 participants, 15 of whom had recorded dizziness at baseline prior to starting total diet replacement, with nine of these on antihypertensive or diuretic medications.

**Conclusions/interpretation:**

Replacing antihypertensive medications with a 3470 kJ/day (830 kcal) diet to induce weight loss reduces BP substantially and may increase mild dizziness. It is safe to stop antihypertensives, but BP should be monitored regularly, particularly for those taking two or more antihypertensives, as over two-thirds will require reintroduction of some medications. Long-term support to maintain weight loss is vital.

**Trial registration:**

ISRCTN registry, number 03267836.

**Graphical abstract:**

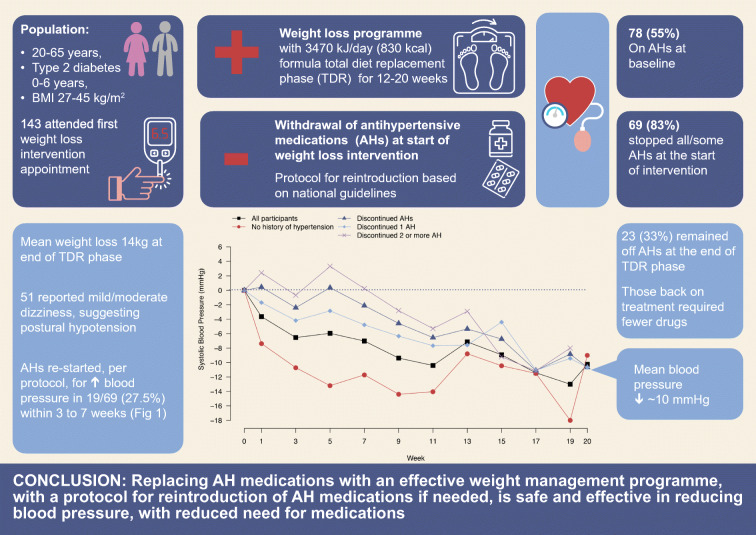

**Supplementary Information:**

The online version contains peer-reviewed but unedited supplementary material available at 10.1007/s00125-021-05471-x.

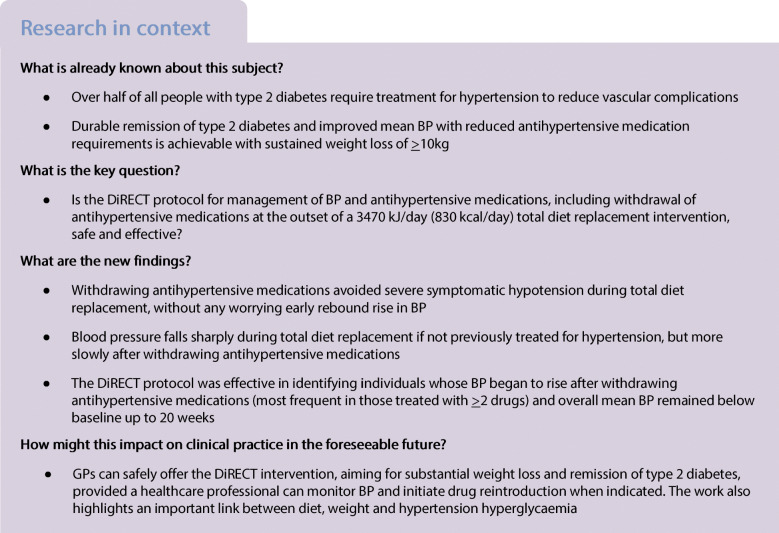



## Introduction

Until recently, type 2 diabetes was viewed as a discrete endocrine disease, and its management was largely limited to prescribing medications that lower blood glucose and HbA_1c_, with a target, representing ‘good control’ of HbA_1c_ < 53 mmol/mol (7%). It is well established that lowering blood glucose and HbA_1c_ will delay or prevent microvascular complications [1–3], but life expectancy for people with type 2 diabetes remains reduced despite best practice treatments directed at clinical guideline targets [4]. The excess morbidity and early mortality are importantly accounted for by vascular complications inherent in the underlying metabolic syndrome, and particularly related to high BP, which commonly accompanies type 2 diabetes [5].

Our understanding of type 2 diabetes is changing, as evidence accumulates that it is primarily a nutritional disease process. It is driven by weight gain; in susceptible or predisposed individuals, body fat accumulates in ectopic sites, specifically liver, pancreas and muscle including heart muscle. A linked underlying genetic or epigenetic predisposition underpins the development of hypertension and dyslipidaemia, the main features of the metabolic syndrome, and also microalbuminuria and hyperuricaemia, commonly in the same individuals. About 85% of people with type 2 diabetes have or will develop hypertension, which requires treatment under current guidelines (systolic BP [SBP] ≥140 mmHg, diastolic BP [DBP] ≥90 mmHg) [6, 7].

As the vascular complications of type 2 diabetes, which still affect most patients, are strongly predicted by BP, clinical guidelines now stress the need for effective antihypertensive treatments [7]. Unfortunately, some medications to treat high BP can aggravate glucose tolerance [8–10] and some anti-obesity medications used in the past to treat type 2 diabetes can elevate BP [11, 12]. All the features of cardiometabolic conditions, including both type 2 diabetes, dyslipidaemia and hypertension, are improved by weight loss [13–15]. However, clinicians and guidelines have been reluctant to recognise weight loss as an effective alternative treatment for hypertension in individuals who are overweight, or to consider a therapeutic trial of withdrawing antihypertensive drug treatment when effective weight management is provided.

The present study is a secondary analysis of the changes in BP and in antihypertensive medication use during the initial total diet replacement (TDR) phase in the intervention arm of the Diabetes Remission Clinical Trial (DiRECT), after a planned withdrawal of all antihypertensive and diuretic medications at the start of an evidence-based weight management programme [16]. Our aim was to determine the safety of stopping BP medications as well as the extent of BP change in each group. We also wanted to assess to what extent BP would fall with weight reduction in our non-hypertensive participants. The per-protocol withdrawal of antihypertensive medications in DiRECT was informed by observation of postural hypotension necessitating withdrawal of antihypertensive agents [17] and the subsequent early results of omitting all antihypertensive agents [18].

## Methods

### DiRECT study design and participants

The detailed protocol, methods and baseline clinical characteristics for DiRECT have been published in full [19, 20]. Briefly, DiRECT is a cluster-randomised, open-label clinical trial with primary care practice as the unit of randomisation. General practices representing populations with wide ranges of social and geographic features across Scotland and in the Tyneside region of England were invited to participate. Practices agreeing to participate were randomised to intervention or control. The main inclusion criteria for participants were type 2 diabetes diagnosed within 6 years, with most recent HbA_1c_ ≥ 48 mmol/mol (6.5%), or ≥43 mmol/mol (6.1%), if on anti-diabetes medication, aged 18–65 years and BMI 27–45 kg/m^2^. The upper BMI limit was to allow magnetic resonance studies. Intervention and control participants continued to receive their usual diabetes care (including that related to hypertension) under current NHS guidelines and standards from NICE in England and SIGN in Scotland [21, 22]. Ethical approval was secured on 24 January 2014. All participants provided written informed consent.

### Intervention

The intervention was an evidence-based weight management programme. Counterweight-Plus [16, 23] was delivered in the participants’ own general practice by the practice nurse or local dietitian who received 12 h training and ongoing mentoring in the programme from the study research dietitians. Weight loss was initiated by TDR using a low-energy, 3452–3569 kJ/day (825–853 kcal/day) formula diet for 12 weeks, extendable up to 20 weeks to allow for planned breaks and participant wishes, followed by stepped food reintroduction (FR) over 2–8 weeks, and a structured programme with monthly visits to support long-term weight loss maintenance (WLM).

To avoid postural hypotension during weight loss [18, 24, 25], the DiRECT protocol adopted the approach used in the Counterbalance study [18] that all antihypertensive (including diuretic) medications were withdrawn on commencement of TDR, irrespective of baseline BP or number of antihypertensive medications being prescribed. Advice to reduce dietary sodium was reinforced [19]. Multi-purpose medications such as beta-blockers were continued if they had been prescribed for an indication other than hypertension.

Blood pressure was measured (sitting and rested) at each intervention visit (1 week after start of TDR then every 2 weeks until the end of FR and monthly thereafter). A pre-specified protocol (Table 1) based on current national clinical guidelines [7, 22] was applied for medication reintroduction if SBP exceeded 165 mmHg (during weeks 1 or 2 of TDR) or 140 mmHg subsequently. There was no pre-planned testing for postural hypotension by lying/standing BP.

**Table 1:** Protocol for reintroduction of antihypertensive medications

1. In first 2 weeks after stopping antihypertensives and diuretics: If SBP is over 165 mmHg on repeated measurement, restart one drug, as below.	
2. Thereafter, if SBP is >140 mmHg, restart one drug as below.	
3. Increase dose weekly to achieve target.	
4. If SBP remains >140 mmHg on the first drug, add a second drug, as below	
5. Increase dose weekly to achieve target.	
6. Repeat as necessary with third, fourth or more drugs (increasing each to maximum dose).	
*Order of reintroduction of previously used drugs*	
1. ACE inhibitors (ramipril, lisinopril, perindropril, etc.)	
2. Angiotensin receptor blockers (irbesartan, candesartan, etc.)	
3. Thiazide type (bendroflumethazide, indapamide, etc.)	
4. Spironolactone	
5. Calcium channel blocker (nifedipine, amlodipine, etc.)	
6. Beta blocker (atenolol, labetolol, etc.)	
7. Alpha blocker (doxazosin, prazosin)	
8. All others	

For participants whose glycaemic control deteriorated, anti-diabetes medications were also reintroduced [19]; medications such as GLP-1 agonists that reduce BP were not used. A checklist of possible side effects related to TDR, including dizziness, was completed at each study visit.

### Statistical analysis

Post hoc analysis was performed using paired *t* tests for changes in BP and weight for intervention participants who commenced TDR (*n* = 143), for the subset with known hypertension who had previously been prescribed antihypertensive medications and discontinued them when starting TDR (*n* = 69), for those stopping 1 (*n* = 33) or ≥2 drugs (*n* = 36) at commencement of TDR and for those with no history of hypertension.

To model changes over time, linear mixed effects regression models were used to predict changes in SBP and DBP and changes in weight from week of visit, treating week of visit as a categorical variable. All models adjust for baseline value of the outcome, age, sex, study centre, practice list size and a random patient effect. Analyses were carried out using SPSS (V24) and R (V3.6.2).

Participants for whom the 12- and/or 24-month remission status was not known were assumed not to have achieved remission, in line with the primary analysis of the study. Missing values in other variables were not imputed.

## Results

### Baseline characteristics (at recruitment)

**Fig. 1:**
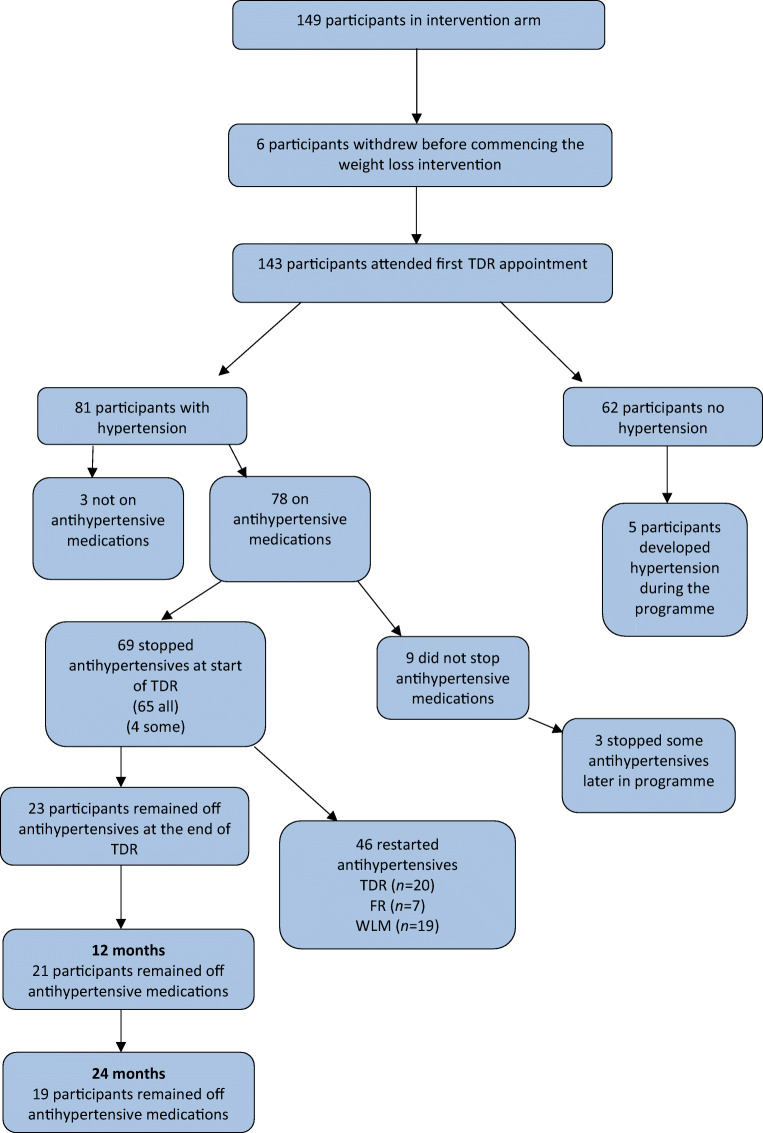
Study participants

One hundred and forty-three participants (79 male, 64 female) from 23 practices allocated to intervention attended the first TDR appointment (visit 1, week 0) and commenced TDR (Fig. 1).

Just over half of all participants (*n =* 81) in the intervention arm had diagnosed hypertension (Table 2). The majority (96%) of those with diagnosed hypertension were on antihypertensive medications, with 54% (44/81) on two or more medications. Baseline weight and BP were lower in those with no history of hypertension in comparison with those with a history of hypertension and to the group as a whole. Baseline BP in those prescribed antihypertensive medications at recruitment (*n* = 78) varied slightly according to the number of medications prescribed:

- One medication (*n* = 34), 136.5 (SD 17.7) mmHg systolic, 85.7 (SD 11.6) diastolic
- Two medications (*n* = 28), 141.4 (SD 21.5) mmHg systolic, 87.0 (SD 11.1) diastolic
- Three medications (*n* = 10), 123.0 (SD 10.7) mmHg systolic, 82.8 (SD 5.7) diastolic
- Four medications (*n* = 6) 135.8 (SD 19.2) mmHg systolic, 82.4 (SD 9.1) diastolic

**Table 2:** Baseline characteristics (at recruitment) of the DiRECT intervention participants who commenced TDR

Variable	All (*n* = 143)	No history of hypertension (*n* = 62)	History of hypertension (*n* = 81)	Discontinued some (4) or all (65) antihypertensive medications at baseline (*n* = 69)	Did not discontinue antihypertensive medications at baseline (*n* = 9)
Age	52.9 ± 7.5	52.1 ± 7.7	53.6 ± 7.4	53.6 ± 7.2	53.4 ± 10.2
Male	79 (55.2)	33 (53.2)	46 (56.8)	42 (60.9)	3 (33.3)
Female	64 (44.8)	29 (46.8)	35 (43.2)	27 (39.1)	6 (66.7)
Diabetes duration (years)	3.0 ± 1.6	2.9 ± 1.6	3.0 ± 1.6	3.0 ± 1.6	3.9 ± 1.7
Weight (kg)	100.9 ± 16.7	99.1 ± 17.5	102.3 ± 16.1	102.7 ± 16.6	100.1 ± 14.7
BMI (kg/m^2^)	35.1 ± 4.5	35 ± 4.6	35.1 ± 4.4	35.0 ± 4.4	35.6 ± 4.8
SBP (mmHg)	132.9 ± 17.4	127.8 ± 13.6	136.7 ± 19.0	135.8 ± 18.1	141.2 ± 26.6
DBP (mmHg)	84.5 ± 10.0	82.8 ± 8.8	85.7 ± 10.8	85.2 ± 9.6	87.5 ± 16.8
Number of antihypertensive drugs prescribed at baseline					
0			3 (3.7)	0	0
1			34 (42.0)	30 (43.5)	4 (44.4)
2			28 (34.6)	23 (33.3)	5 (55.6)
3			10 (12.3)	10 (14.5)	0
4			6 (7.4)	6 (8.7)	0

Data presented as mean ± SD or *n* (%)

### Retention

**Fig. 2:**
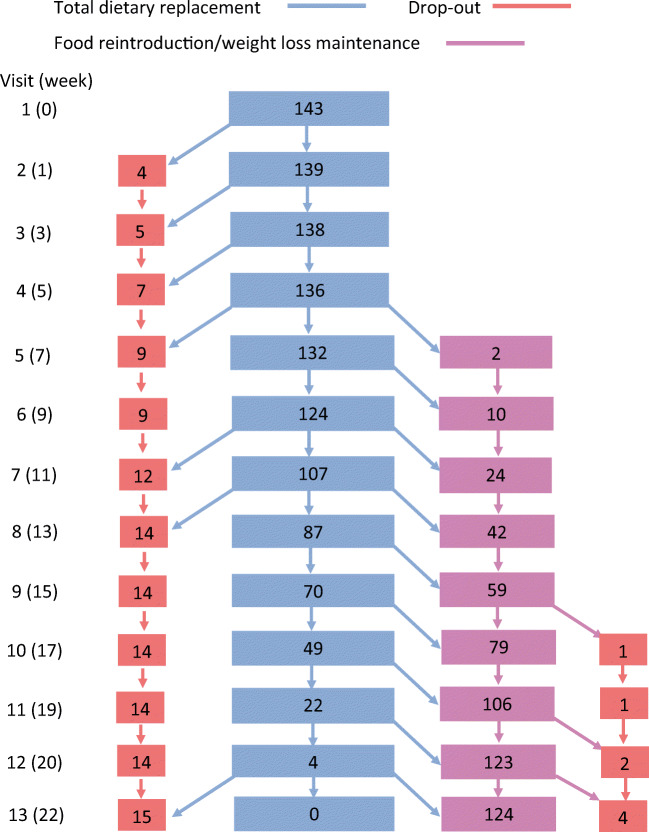
Number of participants continuing in TDR, moving to FR/WLM and withdrawing between visit 1 and visit 12 (inclusive)

Nineteen participants withdrew from the intervention from visit 1 to 12 (inclusive) (Fig. 2). Four attended only the first TDR appointment. Of those who withdrew, 15 were still in the TDR phase of the study. Mean baseline BP of these 15 was 122.8 (SD11.9) mmHg systolic, 82.8 (SD 9.2) mmHg diastolic, with five on antihypertensive medications.

### Discontinuation of antihypertensive medications

All antihypertensive medications were discontinued at the start of TDR, as per DiRECT protocol, in the majority of participants on treatment for hypertension (65/78; 83%). In four participants (5%) only some of the prescribed medications were discontinued (Fig. 1), the reasons given for continuing medications were ‘GP decision’ (2), CHD (1), unknown (1).

In 9 (11.5%) participants none of the antihypertensive medications were stopped on commencing TDR. Their baseline characteristics are shown, compared with the whole group, in Table 2. The reasons given for not stopping antihypertensive medications were: heart failure (1), ‘high BP’ (5), patient reluctant (1), and reason unknown (2). For three of these nine participants, antihypertensive medications were subsequently discontinued during TDR, as BP control improved with weight loss.

### Weight loss

**Fig. 3:**
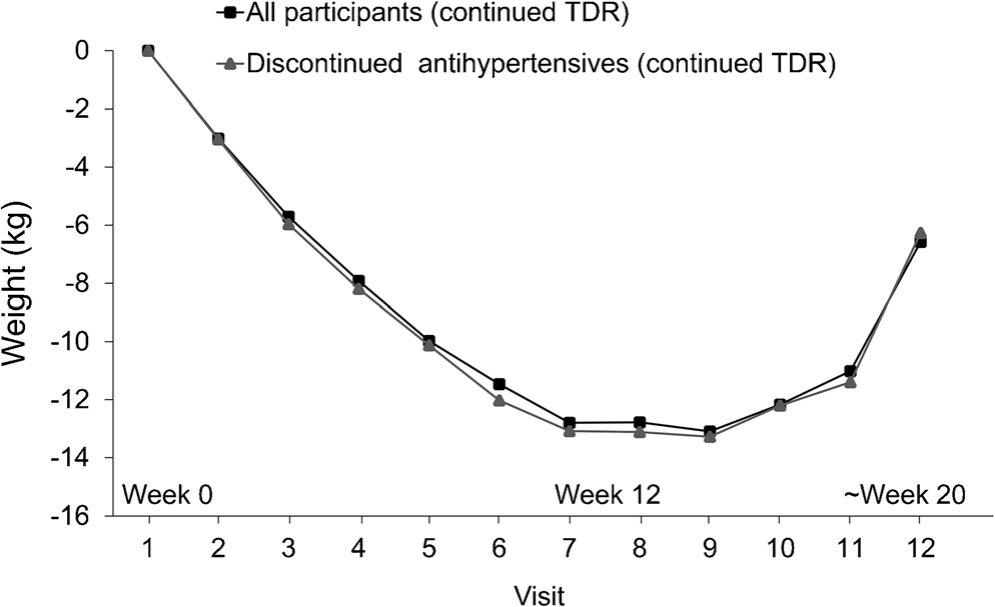
Mean changes in body weight during TDR from baseline (visit 1, week 0) for the whole intervention group (*n* = 143) and for those who discontinued antihypertensive medications (*n* = 69). Numbers decline as participants move to FR or withdraw (TDR continued beyond 12 weeks for small numbers with poorer adherence, whose weight losses were smaller)

Similar significant weight losses were seen during TDR in the intervention group as a whole and those who withdrew antihypertensives (Table 3, Fig. 3, electronic supplementary material [ESM] Table 1). Weight loss was similar for those with no history of hypertension and who discontinued 1 and ≥2 antihypertensives (ESM Fig. 1).

**Table 3:** Mean changes in body weight (kg) from TDR visit 1 (week 0) in those continuing in TDR

Week	All participants (*n* = 143)			Discontinued antihypertensive medications (*n* = 69)		
	n		*p* value	n		*p* value
1	139	−3.01 ± 1.58	<0.0001	69	−3.04 ± 1.56	<0.0001
3	138	−5.71 ± 2.48	<0.0001	69	−5.97 ± 2.44	<0.0001
5	136	−7.92 ± 3.19	<0.0001	69	−8.19 ± 3.26	<0.0001
7	132	−9.98 ± 4.27	<0.0001	69	−10.13 ± 3.96	<0.0001
9	123	−11.46 ± 4.60	<0.0001	65	−12.02 ± 4.73	<0.0001
11	107	−12.80 ± 5.45	<0.0001	58	−13.08 ± 5.76	<0.0001
13	86	−12.78 ± 5.88	<0.0001	48	−13.11 ± 6.87	<0.0001
15	70	−13.09 ± 5.80	<0.0001	40	−13.28 ± 6.47	<0.0001
17	49	−12.17 ± 4.97	<0.0001	29	−12.20 ± 4.96	<0.0001
19	22	−11.02 ± 4.80	<0.0001	12	−11.40 ± 4.90	<0.0001
20	4	−6.57 ± 4.51	0.062	3	−6.27 ± 5.48	0.186

Data presented as mean ± SD

Numbers decline as participants move to the FR phase or withdraw

### Changes in BP

**Fig. 4:**
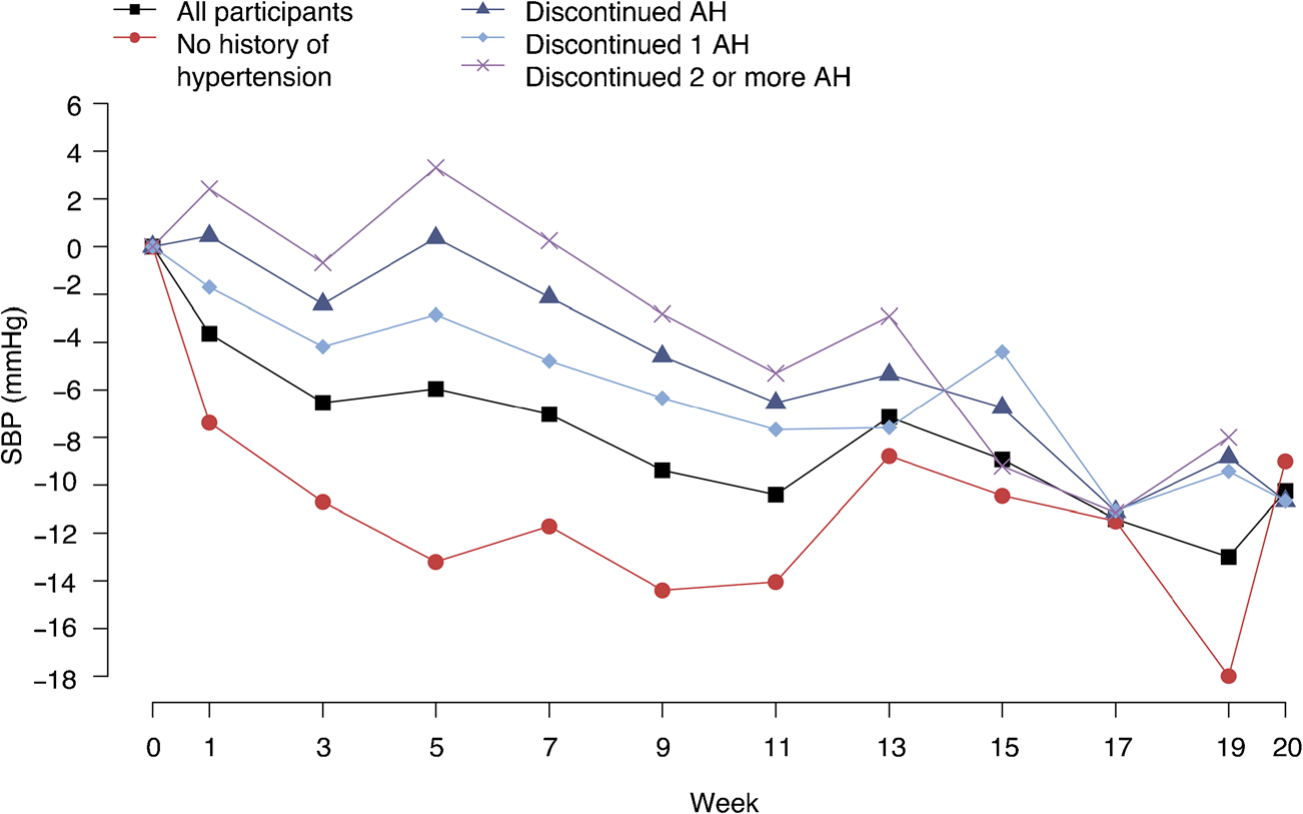
Changes in SBP (mmHg) while continuing in TDR for all participants, those with no history of hypertension, all who discontinued antihypertensive medications, and those who discontinued one and ≥ two antihypertensive medications. AH, antihypertensive medication

In the intervention group as a whole (*n* = 143) significant reductions in mean BP were seen during TDR: SBP fell significantly from week 1 (−3.6 [14.2] mmHg *p* = 0.003), DBP from week 3 (−1.9 [8.7] mmHg, *p* = 0.009) (Fig. 4, ESM Table 2). The falls in BP through TDR were immediate and quite substantial for those with no history of hypertension (Fig. 4, ESM Table 3). In those previously treated for hypertension, and who discontinued antihypertensives, there were no significant changes in BP until visit 6 (~week 9), when both SBP and DBP fell: −4.5 (15.9) mmHg, *p* = 0.03 and −2.5 (9.5) mmHg, respectively (*p* = 0.03) (Fig. 3, ESM Table 2). Changes in BP were slower for those who stopped two or more antihypertensives at the start of TDR (Fig. 4, ESM Table 2). The lowest BP recorded at study visits during TDR was 100/70 mmHg in one participant who had previously been hypertensive, and 95/58 mmHg for one participant without previously treated hypertension. These low BP measurements occurred at visit 6, around week 9 of TDR.

In linear mixed effects regression models predicting change in SBP from week 1, there was a significant difference between those who were and were not on antihypertensive treatment at baseline. Mean SBP was higher by 10.6 mmHg (95% CI 7.2, 14.0; *p* < 0.0001) and mean DBP higher by 4.2 mmHg (95% CI 2.1, 6.3; *p* < 0.0001) for those on antihypertensive treatment at baseline (Table 4). Whether or not a participant was still on TDR was not a significant predictor of SBP or DBP (*p* = 0.1350 and *p* = 0.4868, respectively). There was no significant interaction with treatment visit (*p* = 0.45 for SBP, *p* = 0.73 for DBP), which means the trajectory is similar between both groups.

**Table 4:** Linear mixed effect regression models predicting change in weight, SBP or DBP from baseline value, treatment visit, whether or not the patient was on antihypertensive medication at baseline, and whether or not the participant is still in TDR, adjusted for age, sex, study centre and practice list size and a random effect for patient

Week	Weight		SBP		DBP	
	Adjusted mean change (95 CI%)	*p* value	Adjusted mean change (95 CI%)	*p* value	Adjusted mean change (95 CI%)	*p* value
1	−5.40 (−7.17, −3.62)	<0.0001	−9.52 (−14.08, −4.95)	0.0001	−3.57 (−6.42, −0.71)	0.0161
3	−8.07 (−9.84, −6.30)	<0.0001	−12.46 (−17.03, −7.88)	<0.0001	−4.63 (−7.49, −1.77)	0.0018
5	−10.25 (−12.03, −8.48)	<0.0001	−11.53 (−16.10, −6.95)	<0.0001	−4.48 (−7.34, −1.62)	0.0025
7	−12.27 (−14.04, −10.50)	<0.0001	−13.01 (−17.56, −8.44)	<0.0001	−5.39 (−8.24, −2.54)	0.0003
9	−13.67 (−15.43, −11.91)	<0.0001	−14.40 (−18.92, −9.87)	<0.0001	−6.81 (−9.63, −3.98)	<0.0001
11	−14.83 (−16.58, −13.08)	<0.0001	−15.01 (−19.48, −10.53)	<0.0001	−6.38 (−9.17, −3.59)	<0.0001
13	−15.27 (−17.01, −13.54)	<0.0001	−12.99 (−17.39, −8.58)	<0.0001	−5.46 (−8.21, −2.71)	0.0001
15	−15.82 (−17.55, −14.10)	<0.0001	−14.85 (−19.21, −10.49)	<0.0001	−6.10 (−8.81, −3.39)	<0.0001
17	−15.94 (−17.66, −14.23)	<0.0001	−16.28 (−20.60, −11.95)	<0.0001	−6.83 (−9.52, −4.14)	<0.0001
19	−15.67 (−17.38, −13.96)	<0.0001	−14.38 (−18.69, −10.08)	<0.0001	−5.93 (−8.60, −3.25)	<0.0001
20	−15.43 (−17.15, −13.72)	<0.0001	−14.45 (−18.77, −10.14)	<0.0001	−5.65 (−8.33, −2.96)	0.0001
*p* for week overall		<0.0001		<0.0001		0.0008
On antihypertensives at baseline	−0.62 (−1.97, 0.72)	0.3638	10.60 (7.22, 13.98)	<0.0001	4.20 (2.08, 6.32)	0.0001
In TDR	0.73 (0.17, 1.30)	0.0113	−1.44 (−3.33, 0.45)	0.1350	−0.43 (−1.66, 0.79)	0.4868

Interactions of visit and baseline antihypertensive medication, visit and being in TDR and the three-way interaction of visit, being in TDR and baseline antihypertensive medication were not significant and have been removed from the model

Number of participants contributing to models: 138 for weight change, 137 for SBP and DBP

Analysing all intervention patients who started TDR in a linear mixed effects regression model, weight change was a significant predictor of change in BP (with a decrease of 0.53 [0.41, 0.65] mmHg in SBP and 0.34 [0.26, 0.41] mmHg in DBP per kg lost, *p* < 0.0001 for both) (Table 5). The association did not vary significantly by visit (*p* for interaction of weight change with visit = 0.107 for SBP and 0.656 for DBP, *p* for visit number = 0.2871 and 0.3579), so visit number was therefore removed from the model.

**Table 5:** Predicting BP from weight change in all intervention patients. Linear mixed effects regression model predicting change in BP from change in weight, being on antihypertensive medication at baseline and being in TDR phase, adjusting for age, sex, baseline weight, baseline BP, centre, practice list size and a random effect for patient

Predictor	SBP	*p* value	DBP	*p* value
Weight change	0.53 (0.41, 0.65)	<0.0001	0.34 (0.26, 0.41),	0.0001
In TDR	−2.20 (−3.51, −0.89)	0.0010	−1.38 (−2.22, −0.53),	0.0015
Antihypertensive medication at baseline	3.60 (−1.12, 8.31)	0.1349	3.11 (0.18, 6.04)	0.0375

Data are presented as adjusted mean change with 95% CI

### Postural hypotension/dizziness

Fifty-one participants (36%) reported experiencing dizziness, potentially suggesting postural hypotension, on at least one occasion during the 12–20-week TDR phase, of whom 23 (45%) had been on antihypertensive medications and two had continued them. In most participants (*n* = 42/51, 82%), the reported dizziness was mild, not interfering with daily activities. Only two participants reported experiencing severe dizziness, one of whom had a history of hypertension and had discontinued all antihypertensive medications at the start of TDR. Recorded BP at the time severe dizziness was reported (week 3) was 150/110 mmHg and the participant was recommenced on antihypertensive medication.

Dizziness was reported at baseline (visit 1), before the start of TDR, in 15 participants, of whom nine were taking antihypertensive medications, and two took diuretics which were stopped at the start of TDR. Dizziness, which recurred in most of these 15 participants, could not be attributed fully to the TDR intervention.

### Recommencement of antihypertensive medications

In two-thirds of the participants who discontinued antihypertensive medications (46/69; 66.6%) antihypertensive medications had to be recommenced using the DiRECT reintroduction protocol (Table 1). Antihypertensive drug reintroduction was during TDR for 20/69 participants (29%), mainly among those previously prescribed ≥2 antihypertensives at baseline (14/20). For one participant, this was because of withdrawal from the trial. Changes in BP for the 19 participants who recommenced antihypertensives during TDR because of increases in BP are shown in ESM Table 4. For 11 participants, antihypertensive medications were recommenced around week 3 of TDR, with mean weight change −4.6 (SD 2.1) kg (*p* < 0.001)**,** mean SBP 158.7 (11.7) mmHg and mean DBP 94.0 (10.4) mmHg. In 26 participants, BP remained acceptable throughout TDR, and antihypertensive medications remained withheld, but were recommenced because BP rose later on (Fig. 1). Among those who restarted antihypertensives during WLM, seven did so in year 1 and 12 in year 2.

Of the 46 participants who recommenced antihypertensive medications, around one-third (16/46; 34.8%) had stopped these at 12 months (Table 6). Blood pressure remained well controlled, on fewer medications, at both 12 and 24 months (Table 6).

**Table 6:** BP and number of antihypertensive drugs prescribed at baseline, 12 and 24 months for participants who restarted antihypertensive medications during TDR, FR or WLM and for all participants

Number of antihypertensive drugs prescribed at baseline	Participants who had to recommence antihypertensive medications (*n* = 46)			All (*n* = 143)		
	Baseline	Year 1	Year 2	Baseline	Year 1	Year 2
0	0	16 (34.8)	2 (4.3)	65 (45.5)	97 (67.8)	81 (56.6)
1	15 (32.6)	16 (34.8)	24 (52.2)	34 (23.8)	29 (20.3)	37 (25.9)
2	17 (37.0)	11 (23.9)	11 (23.9)	28 (19.6)	14 (9.8)	16 (11.2)
3	8 (17.4)	3 (6.5)	8 (17.4)	10 (7.0)	3 (2.1)	8 (5.6)
4	6 (13.0)	0	1 (2.2)	6 (4.2)	0 (0)	1 (0.7)
BP (mm/Hg)	(*n* = 46)	(*n* = 41)	(*n* = 36)	(*n* = 143)	(*n* = 127)	(*n* = 112)
Systolic	138.8 ± 19.8	141.8 ± 13.0	136.7 ± 12.9	132.9 ± 7.5	133.1 ± 16.4	130.3 ± 13.6
Diastolic	86.6 ± 9.3	87.2 ± 10.4	85.7 ± 7.0	84.5 ± 10.1	83.6 ± 9.5	81.6 ± 8.5

Data presented as mean ± SD or *n* (%)

Twenty-three (33.3%) of the 69 participants who discontinued antihypertensive medications at TDR baseline remained off the discontinued antihypertensive and diuretic medications through to the end of TDR (Fig. 1). Changes in BP while on TDR are shown in ESM Table 4.

Mean weight loss by week 20 in this group was −16.6 (SD 7.8) kg, SBP 129.5 (SD 14.9) mmHg, at which time most participants were in FR (*n* = 16; 70%). Of these 23, 19 were able to remain off medications at 2 years.

Of the 53 patients in the intervention group who were in remission after 2 years, and with a mean weight loss of 11.4 kg, 31 had been hypertensive at baseline. Of these 31, 27 had all their medication stopped, one had some medication stopped and three had not had their medication stopped at baseline. Of these 27, 12 remained off antihypertensive medication at the end of year 2.

## Discussion

Not all people prescribed antihypertensive drugs need to remain on them indefinitely. Indeed, a systematic review of 66 published studies reporting on withdrawal of antihypertensive drugs found that about 40% of people remain below the treatment threshold at 1 year, and 26% of over 1000 individuals remained normotensive and off medication for 2 years or longer, without evidence for adverse clinical outcomes [26]. However, the strong association between hypertension, affecting 50% or more people with type 2 diabetes [27, 28] as related features of the metabolic syndrome, may point to greater need to continue antihypertensive medication. It is well known that weight loss usually reduces BP, often substantially, as shown for our participants without previous antihypertensive treatment (ESM Table 3). Previous studies have reported reductions in use of antihypertensive drugs with weight loss, but their protocols have not previously included a proactive therapeutic trial of withdrawing medication. Discontinuation of antihypertensive medications at baseline was included in the DiRECT protocol primarily as a safety measure to avoid symptoms and injuries from postural hypotension, (a condition responsible for around 30,000 UK hospital admissions annually [29]). Although modern antihypertensive drugs are effective, it is well known that adherence to prescriptions for both diabetes and hypertension can be poor [30, 31] and the potential to be able to stop both antihypertensive and glucose-lowering medications legitimately was a major practical motivation for participants’ achieving substantial weight loss and remission of diabetes [32, DiRECT unpublished data, paper in preparation].

Withdrawing antihypertensive medications was initially a concern for some GPs in practices participating in DiRECT, uncertain whether weight loss would be achieved, or could provide good alternative treatment for hypertension. However, the results from Counterbalance, showing a fall in BP despite withdrawal of antihypertensive medications, were persuasive [18].

In the event, after withdrawing antihypertensive medications on commencing TDR, no severe postural hypotension was experienced by participants. Importantly, there was no worrying early rebound rises in BP. Indeed, with the combined effects of negative energy balance and weight loss, blood pressures actually fell below the baseline TDR values by about 9 weeks. The decrease in BP on commencing rapid weight loss, with withdrawal of antihypertensive medication, was similar in the community setting of DiRECT to that reported in the research centre-based Counterbalance study [18]. Weight loss was a strong determinant of fall in BP; however, reintroduction of medication was required for about one-third of participants during TDR, despite some weight loss. The protocol in place in DiRECT for monitoring BP and reintroduction of antihypertensive medications [19] allowed these participants to be identified quickly and managed safely. No serious adverse events related to rises in BP occurred.

The ability to provide, within routine primary care, a safe non-surgical intervention to achieve remission of type 2 diabetes without the need for medication is attractive to many people currently living with type 2 diabetes, and at risk of its progressive complications. The DiRECT study has proved that this is possible, with sustained remissions for over 80% at 2 years if weight loss of 10–15 kg is achieved. The present analysis shows a bonus for those individuals who achieved remission, from the high likelihood of being able to withdraw antihypertensive and diuretic medications completely. As with drug treatments, the clinical effects from weight loss do vary between patients, so it is necessary to monitor blood glucose and BP, and respond quickly to any deterioration. This entails a small commitment from either healthcare staff or potentially patients themselves using home monitoring. Applying the simple protocol used in DiRECT for reintroduction of anti-diabetes and/or antihypertensive medications [19] proved safe and effective. The overall effect of the DiRECT intervention at 12 and 24 months was to achieve improvements in mean BP which did not differ significantly between the intervention and the well-controlled control group at 12 months [13] but were significantly lower in the intervention group than in the control group at 24 months [33]. At both 12 and 24 months, fewer participants in the intervention group were being prescribed antihypertensive medications than at baseline (baseline 54% [81/149], 12 months: 32% [47/148], 24 months: 47% [61/129]). This differed significantly from the control group at both time points [13, 33].

It is important that BP continues to be monitored, at least annually along with HbA_1c_, as even without weight regain, BP often rises with age [34].

Those who needed to restart antihypertensive medications were more often those who had been treated with two or more medications at baseline. It is possible that some of these might be at greater risk of postural hypotension if the drugs are not withheld during TDR, and BP monitoring is particularly important for this group. If BP is poorly controlled, and with multiple antihypertensive medications, maintaining some antihypertensive drugs during TDR might be safe, provided that lying and standing BP is checked if there are symptoms suggesting postural hypotension. Non-adherence with antihypertensive medications is reported at 45% overall, and 84% in patients with poorly controlled BP [31]. In some cases, patients adopting new health behaviours may increase their compliance with their prescribed drugs, and the combination with the antihypertensive effect of weight loss can be profound.

The overall effect on BP observed from weight loss in DiRECT was substantial, incorporating both the observed mean fall in BP and the reduced numbers of drugs being prescribed to fewer people, probably of a similar order to that achieved by many of the commonly prescribed antihypertensive medications. There are two separate major mechanisms which probably contribute to the observed reduction in BP. First, there was an acute, relatively large direct effect of the negative energy balance [35], together with the profound decrease in sodium intake on commencing the liquid formula diet which provided approximately 2.0–4.1 g daily, depending on the exact combination of soups and shakes chosen. Baseline salt intake has recently been estimated at 8 g per day in the UK [36, 37]. The decrease in BP following acute reduction of salt intake is greater in hypertensive people [38], although in DiRECT a fall in BP was observed in the whole intervention group. Both the acute and lesser longer-term effects from diet changes can be identified from the data collected during TDR in DiRECT, as shown in Fig. 3. It is well-recognised that for each 1 kg of sustained weight loss, BP falls by about 1 mmHg [39]. The step-change onto a 3470 kJ/day (830 kcal) TDR, necessarily low in all macronutrients albeit *relatively* high in carbohydrate, induced marked reductions in BP. The mechanisms may include metabolic effects, perhaps related to the expected reduction in plasma insulin concentrations for those who adhered to the TDR programme. Plasma insulin was not measured frequently in this primary care study, and specifically not over the first few weeks of TDR The median baseline fasting plasma insulin was only 19.8 mIU/ml (IQR 13.8 to 31.9) (DiRECT unpublished data), so any reduction would be unlikely to account for the large BP effects we observed. For the WLM phase of DiRECT, beyond 3–4 months, participants aimed for energy balance, and there was no restriction in carbohydrate intake. Median fasting plasma insulin at 24 months was 12.9 (7.4 to 20.1) mIU/ml, (DiRECT unpublished data). This very modest fall from baseline, again could not account for the large BP effects observed with sustained weight loss. As well as reduction in sodium consumption, reductions in perivascular ectopic fat might contribute to BP lowering with weight loss, via improved vasocrine signalling [40].

Remission from type 2 diabetes is a highly desired goal for people currently living with diabetes [41], and the intervention is highly cost-effective, indeed cost saving, for healthcare as well as increasing Quality-Adjusted Life Years [42]. The conclusion from this secondary analysis of DiRECT data is that a therapeutic trial of replacing antihypertensive medications with an effective weight management programme to achieve marked negative energy balance and rapid weight loss, and with regular BP monitoring and the DiRECT antihypertensive reintroduction protocol, was safe with no worrying rises or falls in BP. It was not possible to identify reliably at baseline the patients who would fail to lose weight or those whose BP responded less well to weight loss. The present analysis used measured data, following standard measurement procedures, so is unlikely to be affected by bias. We conducted the analyses with appropriate adjustments to avoid confounding. The sample studied was very typical of the type 2 diabetes population within 6 years of diagnosis and with typical prevalence of hypertension that was well controlled at baseline. Participants were not selected for high BP, so regression to the mean is not a factor behind the observed changes. The DiRECT control group, with no alterations in routine management, had similar BP to the intervention group at baseline, and these did not change significantly at 12 months [12]. The results can therefore reasonably be extrapolated to the wider type 2 diabetes population.

## Supplementary Information

ESM (PDF 384 kb)

## Data Availability

Once the data collection and planned analyses of DiRECT are complete (expected 2024), anonymised participant level data will be shared on reasonable request. The study protocol and primary results of DiRECT have been published, using a pre-specified statistical analysis plan, and may be obtained from the corresponding author.

## Abbreviations

DBP: Diastolic BP
DiRECT: Diabetes Remission Clinical Trial
FR: Food reintroduction
NICE: National Institute for Health and Care Excellence
SBP: Systolic BP
SIGN: Scottish Intercollegiate Guidelines Network
TDR: Total diet replacement
WLM: Weight loss maintenance
