# Supplementary material for: Antihypertensive medication needs and blood pressure control with weight loss in the Diabetes Remission Clinical Trial (DiRECT)
Source: Diabetologia. 2021 May 31;64(9):1927–38. doi: 10.1007/s00125-021-05471-x (PMC8382659; doi:10.1007/s00125-021-05471-x)
Supplement: Supplementary file 1 — (PDF 384 kb) [file 125_2021_5471_MOESM1_ESM.pdf]

Electronic Supplementary Material

ESM Table 1 Change in body weight estimated by linear mixed effects regression model predicting change in weight (kg) from visit 1 from visit, adjusting for weight at visit 1, age, sex, centre (Scotland or Tyneside), practice list size (≤5700 or >5700) and a random effect for patient.
Number of participants contributing to model = 139.

	**All in intervention group**		**All continuing on TDR**	
**Week**	**Adjusted mean weight change (95 CI%)**	***p* value**	**Adjusted mean weight change (95 CI%)**	***p* value**
**1**	-5.00 (-6.63, -3.37)	<0.0001	-5.17 (-6.44, -3.89)	<0.0001
**3**	-7.67 (-9.30, -6.04)	<0.0001	-7.84 (-9.11, -6.57)	<0.0001
**5**	-9.85 (-11.48, -8.22)	<0.0001	-10.02 (-11.28, -8.75)	<0.0001
**7**	-11.86 (-13.49, -10.23)	<0.0001	-12.01 (-13.28, -10.74)	<0.0001
**9**	-13.29 (-14.93, -11.66)	<0.0001	-13.53 (-14.81, -12.25)	<0.0001
**11**	-14.51 (-16.14, -12.87)	<0.0001	-14.91 (-16.20, -13.62)	<0.0001
**13**	-15.10 (-16.73, -13.46)	<0.0001	-15.43 (-16.76, -14.11)	<0.0001
**15**	-15.74 (-17.38, -14.11)	<0.0001	-15.96 (-17.31, -14.61)	<0.0001
**17**	-15.98 (-17.62, -14.34)	<0.0001	-15.80 (-17.21, -14.40)	<0.0001
**19**	-15.86 (-17.49, -14.22)	<0.0001	-15.47 (-17.07, -13.86)	<0.0001
**20**	-15.73 (-17.37, -14.09)	<0.0001	-13.31 (-16.02, -10.59)	<0.0001

ESM Table 2: Mean changes in BP (mmHg) while continuing in TDR for all participants, those with no history of hypertension, all who discontinued antihypertensive medications and those who discontinued 1 and ≥ 2 antihypertensive medications (Data expressed as mean and ± SD)

		Mean Changes in BP from TDR visit 1 (week 0)							
**Week of TDR**		**All participants (*n*=143 at week 0)**	***p* value**	**Discontinued antihypertensive medications at start of TDR (*n*=69 at week 0)**	***p* value**	**Discontinued 1 drug at start of TDR (*n*=33 at week 0)**	***p* value**	**Discontinued ≥ 2 drugs at start of TDR (*n*=36 at week 0)**	***p* value**
**1**	SBP	-3.64 ±14.17	0.003	0.45 ±14.37	0.796	-1.70 ±15.43	0.532	2.42 ±13.23	0.281
	DBP	-0.88 ±8.73	0.240	0.16 ±8.81	0.881	-0.91 ±9.72	0.595	1.14 ±7.89	0.392
**3**	SBP	-6.54 ±14.29	<0.0001	-2.40 ±13.93	0.163	-4.18 ±15.56	0.133	-0.68 ±12.11	0.747
	DBP	-1.99 ±8.71	0.009	-0.85 ±9.07	0.445	0.00 ±9.65	1.000	-1.68 ±8.52	0.259
**5**	SBP	-5.96 ±16.73	<0.0001	0.36 ±17.60	0.865	-2.85 ±14.74	0.275	3.31 ±19.60	0.319
	DBP	-1.92 ±9.12	0.016	-0.29 ±10.16	0.813	-2.45 ±9.63	0.153	1.69 ±10.36	0.333
**7**	SBP	-7.02 ±16.07	<0.0001	-2.12 ±15.61	0.267	-4.78 ±16.28	0.107	0.25 ±14.82	0.920
	DBP	-2.97 ±9.67	0.001	-1.82 ±9.08	0.102	-3.38 ±10.15	0.069	-0.44 ±7.90	0.738
**9**	SBP	-9.38 ±15.83	<0.0001	-4.58 ±15.93	0.025	-6.34 ±14.77	0.021	-2.81 ±17.06	0.358
	DBP	-4.43 ±9.74	<0.0001	-2.52 ±9.50	0.038	-3.31 ±10.30	0.079	-1.72 ±8.72	0.273
**11**	SBP	-10.41 ±17.53	<0.0001	-6.54 ±17.69	0.007	-7.67 ±16.07	0.014	-5.30 ±19.57	0.171
	DBP	-4.52 ±10.26	<0.0001	-2.84 ±10.62	0.048	-3.07 ±10.54	0.122	-2.59 ±10.90	0.227
**13**	SBP	-7.14 ±17.82	0.0004	-5.35 ±20.21	0.079	-7.58 ±16.50	0.034	-2.91 ±23.77	0.572
	DBP	-2.56 ±10.03	0.021	-1.37 ±9.92	0.354	-1.67 ±10.88	0.461	-1.05 ±9.00	0.592

Week of TDR		All participants (*n*=143 at week 0)	*p* value	Discontinued antihypertensive medications at start of TDR (*n*=69 at week 0)	*p* value	Discontinued 1 drug at start of TDR (*n*=33 at week 0)	*p* value	Discontinued ≥ 2 drugs at start of TDR (*n*=36 at week 0)	*p* value
		**Mean Changes in BP from TDR visit 1 (week 0)**							
**15**	SBP	-8.93 ±18.96	0.0002	-6.74 ±17.76	0.023	-4.40 ±16.01	0.234	-9.21 ±19.56	0.055
	DBP	-2.06 ±11.52	0.143	-0.54 ±11.79	0.777	0.85 ±12.84	0.770	-2.00 ±10.72	0.427
**17**	SBP	-11.42 ±15.41	<0.0001	-11.11 ±16.46	0.001	-11.07 ±16.36	0.020	-11.15 ±17.24	0.038
	DBP	-4.25 ±12.05	0.018	-5.64 ±12.82	0.028	-2.80 ±14.31	0.461	-8.92 ±10.46	0.010
**19**	SBP	-13.00 ±17.29	0.002	-8.83 ±20.42	0.162	-9.43 ±17.94	0.214	-8.00 ±25.73	0.525
	DBP	-4.50 ±11.61	0.083	-6.33 ±12.01	0.095	-3.57 ±13.66	0.515	-10.20 ±9.20	0.068
**20**	SBP	-10.25 ±9.78	0.127	-10.67 ±11.93	0.262	-10.67 ±11.93	0.262	n/a	n/a
	DBP	-2.00 ±7.53	0.632	-4.00 ±7.81	0.469	-4.00 ±7.81	0.469	n/a	n/a

ESM Table 3 Mean changes in BP during TDR for participants with no history of hypertension. TDR baseline BP (visit 1, week 0) was 134.6 (15.5) mmHg SBP, 82.1 (8.3) mmHg DBP (Data expressed as mean and +SD change from TDR baseline, week 0)

**Week of TDR (n)**		**No history of hypertension (*n*=62 at week 0)**	***p* value**
**1 (59)**	SBP	-7.37 ±12.86	<0.0001
	DBP	-1.17 ±7.81	0.255
**3 58)**	SBP	-10.71 ±13.30	<0.0001
	DBP	-2.83 ±8.66	0.016
**5 (55)**	SBP	-13.20 ±12.54	<0.0001
	DBP	-3.29 (±7.86	0.003
**7 (55)**	SBP	-11.71 ±13.23	<0.0001
	DBP	-3.75 ±10.42	0.010
**9 (51)**	SBP	-14.39 ±12.70	<0.0001
	DBP	-6.06 ±9.95	<0.0001
**11 (43)**	SBP	-14.05 ±17.13	<0.0001
	DBP	-5.58 ±9.44	<0.0001
**13 (36)**	SBP	-8.78 ±14.91	0.001
	DBP	-3.53 ±10.28	0.047
**15 (27)**	SBP	-10.44 ±19.84	0.011
	DBP	-3.00 ±11.08	0.171
**17 (18)**	SBP	-11.50 ±14.08	0.003
	DBP	-0.89 ±10.43	0.722
**19 (10)**	SBP	-18.00 ±11.70	0.001
	DBP	-2.30 ±11.32	0.537
**20 (0)**	SBP	n/a	n/a
	DBP	n/a	n/a

ESM Table 4: Changes in blood pressure for those who restarted antihypertensive medication due to increases in blood pressure while continuing in TDR and those who did not restart antihypertensive medications during TDR
(Data expressed as mean ±SD)

Mean changes in BP (mm/Hg) from TDR visit 1 (week 0)

**Week of TDR**		**Restarted antihypertensive medication while continuing in TDR (*n*=19 at week 0)**	***p* value**	**Did not restart antihypertensive medication while continuing in TDR (*n*=23 at week 0)**	***p* value**
**1**	SBP	4.58 ±13.31	0.15	-2.22 ±12.78	0.41
	DBP	1.05 ±5.49	0.41	-0.96 ±9.12	0.62
**3**	SBP	5.16 ±12.92	0.10	-6.73 ±13.54	0.03
	DBP	2.32 ±7.80	0.21	-0.95 ±7.82	0.57
**5**	SBP	7.37 ±20.70	0.14	-3.30 ±13.71	0.26
	DBP	2.79 ±12.39	0.34	-3.70 ±10.22	0.10
**7**	SBP	2.42 ±15.98	0.52	-2.50 ±13.56	0.40
	DBP	-0.05 ±8.34	0.98	-3.68 ±9.76	0.09
**9**	SBP	-2.67 ±19.92	0.58	-5.81 ±12.51	0.05
	DBP	-2.00 ±9.99	0.41	-3.86 ±9.80	0.09
**11**	SBP	-3.63 ±19.26	0.46	-7.05 ±15.26	0.05
	DBP	-2.75 ±11.90	0.37	-3.68 ±11.37	0.17
**13**	SBP	-0.57 ±23.45	0.93	-7.43 ±13.32	0.06
	DBP	-1.36 ±10.32	0.63	-1.21 ±10.84	0.68
**15**	SBP	-4.42 ±21.92	0.50	-1.46 ±11.31	0.65
	DBP	-1.33 ±12.87	0.73	-1.23 ±14.41	0.76
**17**	SBP	-11.10 ±17.32	0.07	-10.67 ±13.36	0.04
	DBP	-8.00 ±13.25	0.09	-4.78 ±13.90	0.33
**19**	SBP	-5.80 ±25.77	0.64	-15.67 ±9.29	0.10
	DBP	-9.20 ±10.50	0.12	-9.33 ±1.15	0.01
**20**	SBP	n/a		n/a	
	DBP	n/a		n/a	

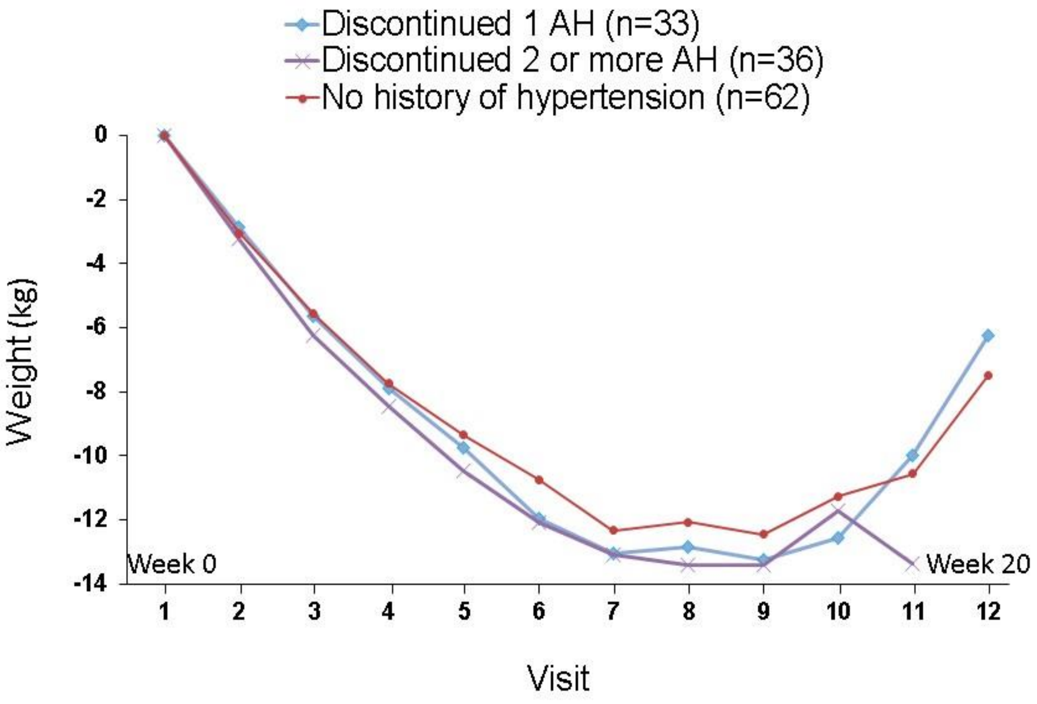

ESM Fig 1: Mean changes in body weight (kg) from TDR visit 1 (week 0) in participants with no history of hypertension and those who discontinued 1 & ≥2 antihypertensive medications while continuing in TDR.
(Number decline as participants move to the Food Reintroduction phase or withdraw)
